# Toxic Epidermal Necrolysis-Like Lesions and Systemic Lupus Erythematosus Possibly Triggered by Sulfasalazine

**DOI:** 10.1155/2016/4501937

**Published:** 2016-07-12

**Authors:** Simon Krabbe, Cigdem Gül, Bjarne Andersen, Niels Tvede

**Affiliations:** ^1^Center for Rheumatology and Spine Diseases, Rigshospitalet, 2100 Copenhagen, Denmark; ^2^Department of Dermatology, Gentofte Hospital, 2900 Hellerup, Denmark; ^3^Department of Rheumatology, Nordsjællands Hospital, 3400 Hillerød, Denmark

## Abstract

This case report describes a patient with arthritis of the large joints, bilateral sacroiliitis, and positive anti-SSA and anti-dsDNA antibody, who received sulfasalazine and shortly thereafter became critically ill. He developed toxic epidermal necrolysis, hemolytic anemia, lymphopenia, markedly elevated ferritin, and muscle wasting. A diagnosis of systemic lupus erythematosus was made, and mycophenolate mofetil and systemic glucocorticoids brought this severe disease under control. Toxic epidermal necrolysis-like lesions and hemophagocytic syndrome have been reported as manifestations of systemic lupus erythematosus. This patient possibly had spondyloarthritis or an undifferentiated connective tissue disease at presentation, and we suggest, based on the timing of events, that sulfasalazine may have acted as a trigger of the severe disease manifestations.

## 1. Introduction

A patient developed a life-threatening disease with toxic epidermal necrolysis- (TEN-) like lesions and systemic lupus erythematosus. Based on earlier case reports with some similarity to this patient's history and disease manifestations, intravenous immunoglobulins, high-dose systemic glucocorticoids, and mycophenolate mofetil were given. Here we report the good outcome of using this strategy for this patient.

## 2. Case Presentation

A 48-year-old man presented with symmetrical arthritis of the wrists, metacarpophalangeal and proximal interphalangeal joints, elbows, and knees. He had had intermittent inflammatory back pain since the age of 40 years. His brother suffered from psoriasis. The physical examination revealed alopecia areata, which he said he had had for a long time, but he had never had psoriasis or other skin manifestations. Radiography showed bilateral sacroiliitis but no erosions of hands or feet. His serology was notable for a high-titer positive anti-SSA antibody of ≥240 kU/L (ref. <7), a low-titer positive anti-dsDNA antibody of 14 kIU/L (<10), and a positive IgM-rheumatoid factor of 19 kIU/L (<10). Anti-CCP and HLA-B27 were negative, and proteinuria was not present. However, he had no symptoms of systemic lupus erythematosus or Sjögren syndrome. A diagnosis of spondyloarthritis was made, and his symptoms improved on NSAID and prednisolone. However, he developed diabetes mellitus, and sulfasalazine was prescribed.

**Figure 1:**
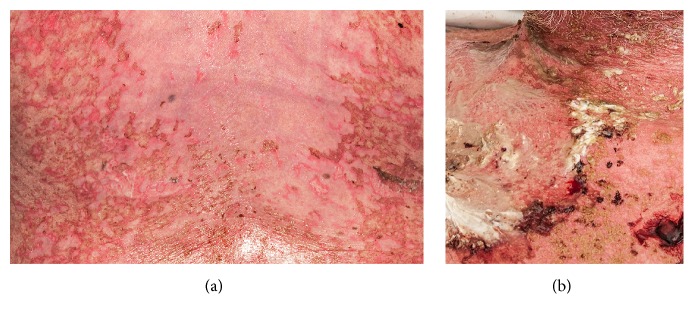
(a) Confluent maculopapular exanthema and bullae and skin erosions of the back. (b) Toxic epidermal necrolysis-like lesions of the anterior chest and neck with bacterial superinfection.

A week after starting sulfasalazine, he presented with erythema of the trunk and neck. A cutaneous drug reaction was suspected, sulfasalazine was immediately stopped, and methotrexate was prescribed instead. Three weeks after stopping sulfasalazine, he was admitted because of fever, cough, dyspnea, loss of appetite, night sweats, and weight loss. The erythema had spread to the extremities with a dark red, confluent maculopapular exanthema, and bullae and denudation of the epidermis in large patches of the back were now observed (Figure 1(a)). MRI of the sacroiliac joints and lumbar spine was consistent with bilateral sacroiliitis but showed no active inflammatory changes. His serology was negative for hepatitis B, hepatitis C, and HIV and not indicative of current infection with EBV, CMV, or parvovirus. Anti-dsDNA antibody had increased to 43 kIU/L, and he had proteinuria 0.7 g/day.

He was managed with intravenous antibiotics and continued on prednisolone 7,5 mg qd, and his skin gradually improved. After 3 weeks, high fever recurred, erythroderma with small pustules on the trunk and extremities was observed, and he developed TEN-like lesions of 10% of body surface area (Figure 1(b)), mostly the back and nates. He was transferred to an intensive care unit due to* Staphylococcus aureus* sepsis and stabilized with intravenous immunoglobulin (IvIg), intravenous antibiotics, methylprednisolone 100 mg qd, and Flamazine cream.

**Figure 2:**
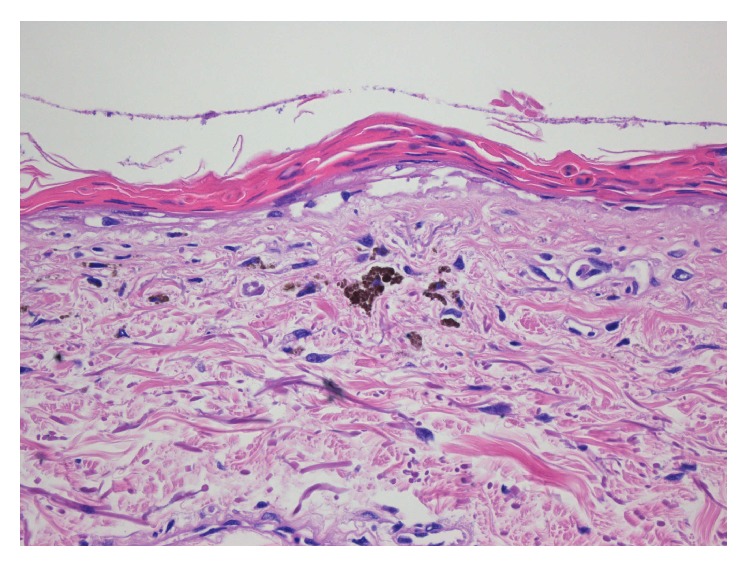
Toxic epidermal necrolysis-like lesion with full thickness necrosis of the epidermis and pigment incontinence (H&E ×40).

After recovering from the sepsis, PET-CT revealed multiple pathologically enlarged lymphatic glands with increased FDG uptake in the neck and axillary and inguinal regions, near porta hepatis, and in the retroperitoneum. A lymph node biopsy showed large areas of histiocytosis but was not diagnostic for dermatopathic lymphadenopathy or hemophagocytosis. Skin biopsy was consistent with TEN-like lesions, showing full thickness necrosis of the epidermis and pigment incontinence (Figure 2). His mucous membranes were not involved. Bone marrow biopsy showed an increased number of myelopoietic precursor cells, sparse erythropoiesis, and slight interstitial infiltration of B and T lymphocytes but no malignancy. Renal biopsy showed focal mesangial hypercellularity and 1 of 15 glomeruli with sclerosis, a faint linear reaction for IgG, and a slight unspecific reaction in IgM and IgA but no complement deposition, and this was not found to be indicative of glomerulonephritis.

## 3. Discussion

A diagnosis of systemic lupus erythematosus (SLE) was made based on the following SLICC criteria: arthritis, proteinuria, hemolytic anemia with a positive direct antiglobulin test after recovering from the sepsis, lymphopenia, high-titer ANA by ELISA and Hep2-cells, and repeatedly positive anti-dsDNA. Hemophagocytic syndrome was highly considered based on the enduring fever, markedly elevated ferritin of 14,900 *μ*g/L (reference interval: 12–300), and a low number of NK-cells, elevated IL-2 receptor of 1500 kU/L (223–710), and CD163 of 10.7 mg/L (0.69–3.86). However, as mentioned, we did not find evidence for this in the biopsies of the bone marrow or lymphatic gland.

Mycophenolate mofetil (MMF) was prescribed based on earlier case reports of successful management of hemophagocytosis in SLE [1–3]. The patient gradually recovered, and one year later he was back to work continuing on MMF and low-dose prednisolone as maintenance treatment. The mortality of TEN has been estimated at 30%, and high doses of intravenous immunoglobulins may be efficacious [4, 5]. IvIg may thus have contributed to the resolution of the TEN-like lesions in this case.

The Naranjo Adverse Drug Reaction probability scale indicated a possible relation between sulfasalazine and the skin and biochemical manifestations. First, life-threatening cutaneous adverse reactions to sulfasalazine have been reported in patients receiving sulfasalazine for inflammatory bowel diseases and psoriatic arthritis [6], and drug-induced SLE has been reported in patients receiving sulfasalazine with sustained autoimmunity even years after stopping the drug [7]. Second, the skin manifestations appeared shortly after sulfasalazine was administered. However, toxic epidermal necrolysis-like skin lesions and hemophagocytic syndrome are possible manifestations of systemic lupus erythematosus, which would be an alternative explanation [8–10]. The lack of mucous membrane involvement is not typical for TEN; however, there were no photodistribution and no histopathologic signs of systemic lupus erythematosus as the cause of the epidermal necrosis [11], and therefore we diagnosed this patient with TEN. In this case, it is peculiar that his skin and fever were ameliorating one month after stopping sulfasalazine, but two months afterwards, his condition suddenly worsened again, suggesting that it was not a direct toxic effect on the skin but an immunological phenomenon and that sulfasalazine may have acted as a trigger.
